# Patient and Parent Perceptions of Disorders of Sex Development Terminology: A Pilot Study

**DOI:** 10.1007/s10508-025-03304-1

**Published:** 2025-11-25

**Authors:** Stephanie Sharp, Linford Williams, Beverly Yashar, Lauren Mohnach, J. Thomas Fitzgerald, Michelle M. Ernst, Tara Schafer-Kalkhoff, Veronica Gomez-Lobo, David E. Sandberg

**Affiliations:** 1 Department of Human Genetics, University of Michigan, Ann Arbor, MI USA; 2 Cancer Risk and Prevention Clinic, Maine Medical Center, Scarborough, ME USA; 3 Division of Medical Genetics, UMPC Children’s Hospital of Pittsburgh, Pittsburgh, PA USA; 4 Differences of Sex Development Clinic, C.S. Mott Children’s Hospital, University of Michigan, Ann Arbor, MI USA; 5 Department of Learning Health Sciences, University of Michigan, Ann Arbor, MI USA; 6 Department of Pediatrics, University of Cincinnati College of Medicine, Cincinnati, OH USA; 7 Division of Behavioral Medicine and Clinical Psychology, Cincinnati Children’s Hospital Medical Center, Cincinnati, OH USA; 8 Division of Endocrinology, Cincinnati Children’s Hospital Medical Center, Cincinnati, OH USA; 9 Pediatric and Adolescent Gynecology, Eunice Kennedy Shriver National Institute for Child Health and Human Development, National Institutes of Health, Bethesda, MD USA; 10 Susan B. Meister Child Health and Evaluation Research Center, Department of Pediatrics, University of Michigan, 2800 Plymouth Road, North Campus Research Complex, Building 16/G035E, Ann Arbor, MI 48109-2800 USA

**Keywords:** Disorders of sex development, Intersex, Terminology, Semantic differential

## Abstract

Diagnostic terminology used for disorders of sex development (DSD) has been controversial even before the introduction of the umbrella term at the 2006 International Consensus Conference on Intersex. Agreement on nomenclature can enhance communication among clinicians, researchers, patients, and their families. However, disagreements over the implications of nomenclature can also result in a proliferation of terms contributing to confusion. The objective of this pilot study was to assess the connotative meaning assigned by adolescent patients with a DSD and parents to the terms used to describe their medical condition. In a sample of 27 adolescents/young adults with a DSD and 22 of their parents/caregivers recruited from three pediatric medical centers in mid-West and mid-Atlantic states, a semantic rating scale measuring self-esteem was used to characterize the connotative meaning ascribed to the person’s specific diagnosis (or anatomical phenotype), the umbrella terms “disorders of sex development,” “intersex,” and “yourself”/“your child.” Youth and parents rated themselves or their children more positively compared to how they rated either the specific diagnosis, the umbrella terms DSD or intersex. The ratings for the latter three terms were generally neutral and did not significantly differ from each other. These pilot findings suggest that neither youth with DSD nor their parents equate the person with their diagnosis. They also evaluated the person more positively than any of the diagnostic or umbrella terms rated. In contrast with previous studies assessing “preferences” for particular terms, this study suggests a novel strategy for assessing the personal “meanings” ascribed to each.

## Introduction

The traditional role of patients as passive recipients of healthcare delivered by healthcare experts has evolved into a patient- and family-centered approach emphasizing the perspectives and values of patients and their caregivers. By concurrently addressing the medical and psychosocial needs of the person, patient-centeredness contributes to improved outcomes, better use of resources, decreased costs and increased satisfaction with care (Gluyas, 2015; Institute of Medicine Committee on Quality of Health Care in America, 2001; Lines et al., 2015). Healthcare providers are encouraged to use language that is preferred, respectful, and empowering to patients. This approach aligns with the patient-centered care movement and is exemplified by the widespread adoption of "person-first" language, which aims to preserve the humanity and individuality of each person (Dunn & Andrews, 2015).

While there has been general acceptance of the concept of patient-centered care, actual implementation has been associated with tensions among subgroups of stakeholders. A keen example of this is seen in terminology for conditions grouped today under the umbrella of “disorders of sex development” (DSD). The key stakeholders in this debate include healthcare providers, medical journal editors, parents of individuals with such conditions, the individuals themselves both as children and as they become adults, and support and advocacy groups. Stakeholders can be driven by divergent perspectives and motivations, such as features of quality healthcare delivery and how these are informed by societal perceptions of and attitudes towards particular diagnoses and those affected.

The International Consensus Conference on Intersex (hereafter referred to as the Consensus Statement) dedicated a major portion of the agenda to addressing these conflicting perspectives and exploring a new nomenclature (Lee et al., 2006). Whereas “intersex,” the umbrella term at the time, “hermaphroditism,” “pseudohermaphroditism” (and its sex-specific qualifiers), and “sex reversal,” were particularly controversial and considered pejorative by many, conference attendees sought to create a terminology that was (1) sensitive to the concerns of patients and families, (2) precise, descriptive, and accurate, particularly in integrating advancing progress in the discovery of molecular genetic etiologies, and (3) robust and consistent as a framework and accommodating of the broad spectrum of phenotypic variation seen in these conditions (Bennecke et al., 2021; Lee et al., 2006). While terminology preferences of individuals and families should certainly guide their use as an important tenet of patient-centered care, it is also necessary to have a standardized baseline for use in initial introductions with families, service-promoting and educational materials, and research publications. Overall, the use of the proposed nomenclature needs to be valued by clinicians and scientists as well as understandable and acceptable to patients and their families.

Based on these guiding principles, DSD was defined as “congenital conditions in which development of chromosomal, gonadal, or anatomic sex is atypical” (Lee et al., 2006). Uptake of the proposed DSD terminology by clinicians and scientists was initially rapid and consistent (Pasterski et al., 2010). Leaders of the intersex advocacy movement in the US and allies from the medical community similarly endorsed the adoption of the new terminology as a means to “label the condition rather than the person” (Dreger et al., 2005). Resistance to the new umbrella term emerged shortly after its introduction (Feder & Karkazis, 2008; Reis, 2007) and has continued unabated. At the heart of the conflict is the view that the word “disorder” in DSD implies a need for surgical or medical interventions, which some regard as a medicalization of biological diversity. In response, but without formal discussions, a shift to the term “differences of sex development” became common and did not require a change in the initialism DSD (e.g., Ahmed et al., 2011; Kreukels et al., 2019; Wiesemann et al., 2010; Wisniewski et al., 2019)). This and the introduction of other compromise umbrella terms did not, however, end the debate and multiple preference surveys followed (Bennecke et al., 2021; Davies et al., 2011; D'Oro et al., 2020; Johnson et al., 2017; Lin-Su et al., 2015; Lundberg et al., 2018).

The term “Intersex” has been reclaimed as a personal and political identity by some to celebrate and reappropriate a medical term, historically viewed as negative, as an identity and expression of the “identity-first” language movement (Dunn & Andrews, 2015; Human Rights Watch, 2017). The most frequently assessed stakeholder groups in studies of terminology preferences have been parents, caregivers, and individuals with a DSD diagnosis, largely via membership in support and advocacy groups (Davies et al., 2011; Johnson et al., 2017; Lin-Su et al., 2015; Lundberg et al., 2018). Fewer studies have recruited participants through medical center settings or clinical research networks (Bennecke et al., 2021; D'Oro et al., 2020; Lundberg et al., 2018). Overall, the results of these studies indicate that most groups do not favor the term “disorder of sex development,” although no other proposed terms have garnered universal support. Of particular note, there is extensive variability in attitudes toward the term DSD, both within and between stakeholder groups. This lack of consistency indicates that there may be unresolved tensions between stakeholder perspectives, which necessitates further investigation beyond preference or dislike of any particular term (Whitehead et al., 2025).

The majority of published surveys assessed the preference for umbrella terms, either the term alone (Davies et al., 2011; Lin-Su et al., 2015), compared directly to another (Davies et al., 2011), or as a list of terms contrasted via ratings scales (Bennecke et al., 2021; D'Oro et al., 2020; Johnson et al., 2017; Miller et al., 2018). While these results have been informative, a deeper understanding of the meaning stakeholders ascribe to these terms is warranted.

Measurement of the implicit perceptions of DSD-related terminology is lacking in the existing literature. The present pilot study utilizes the semantic differential scaling technique in a cohort of adolescents/young adults and their parents/caregivers, recruited from three U.S. medical centers, to measure the connotative meaning of key umbrella terms used in this area of clinical care and clinical research. This study aimed to explore whether the preference for particular terminology by patients and parents, could be reframed as a question of the personal meaning associated with each term. A primary study goal was to compare the meanings ascribed by participants to the terms DSD and intersex. We further sought to compare those ratings to the meanings associated with the patient’s specific diagnosis (or anatomical phenotype). Finally, we sought the appraisals of the person (self- and parent-report) to determine if those judgments corresponded with appraisals of the specific diagnosis or the two umbrella terms, i.e., does the person with a DSD (or parents) perceive an equivalency between the attributes of the person and the various labels (specific or global) for their condition.

## Method

### Participants

**Fig. 1:**
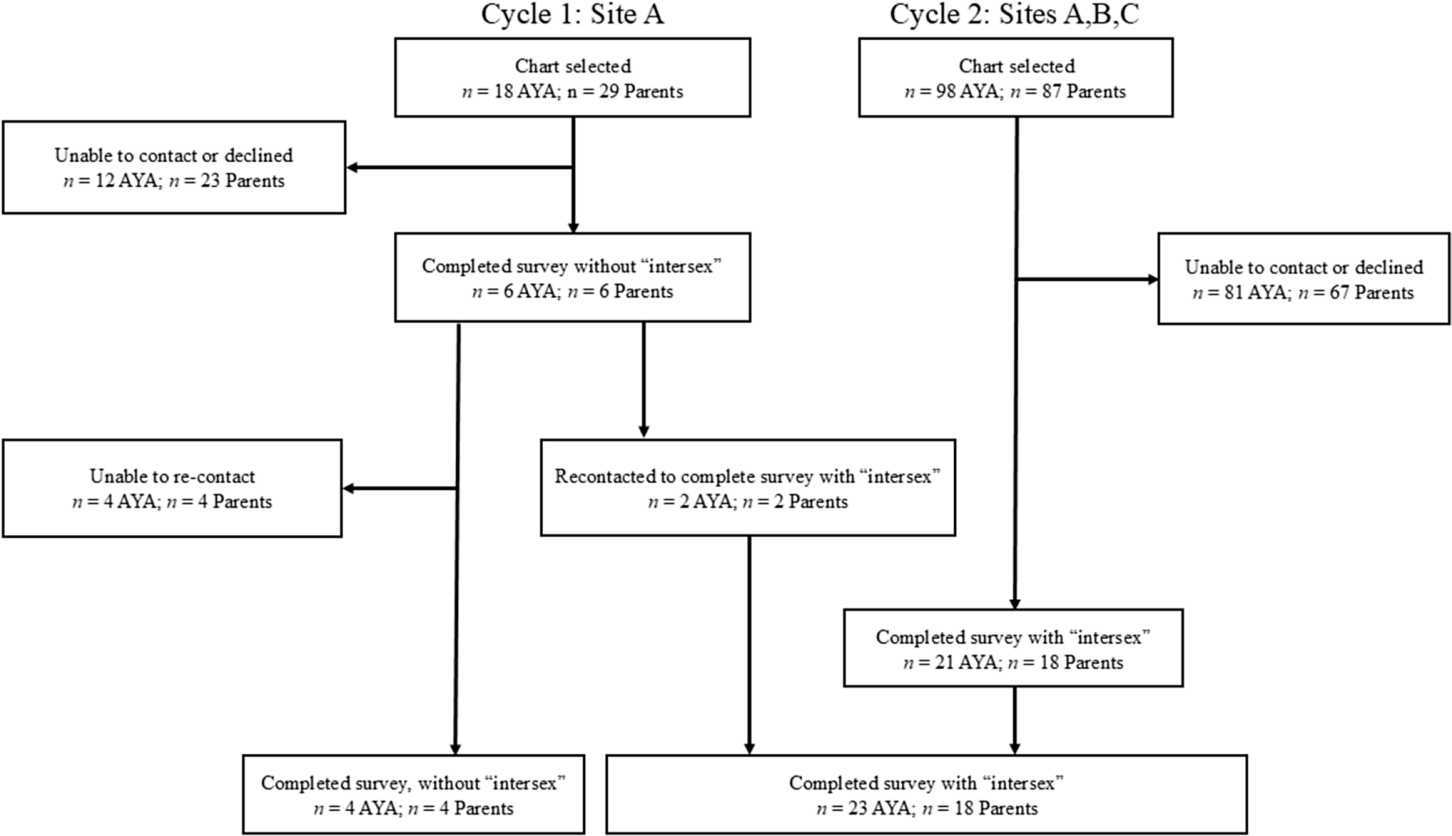
Participant recruitment. Recruitment occurred in two cycles including three medical centers: Cycle 1 involved participants from Site A alone, initially using a preliminary version of the semantic differential which did not include the term “intersex.” Participants were subsequently recontacted to complete the final version of the survey which included “intersex.” Cycle 2 involved recruitment at all 3 sites.

The study surveyed adolescents/young adults (AYA) with a DSD diagnosis and their parents/caregivers. Participants were identified by chart review and recruited at three U.S. pediatric medical centers, each with multidisciplinary DSD clinics in mid-West and mid-Atlantic states. Eligible AYA were between the ages of 12–29 and spoke English as a first language. AYA were, a priori, excluded if information in the medical record indicated a level of developmental delay that would interfere with understanding the survey, or had a diagnosis of either Klinefelter syndrome (47,XXY) or monosomic Turner syndrome (45,X). Klinefelter and Turner syndromes were excluded to sidestep disagreements regarding their categorization as examples of sex chromosome DSD (Wit et al., 2007). Approval of the AYA’s primary subspecialty physician was required prior to contact. A total of 116 total AYA were identified as eligible from the three medical centers (Fig. 1).

Twenty-seven AYA and 22 parents/caregivers completed the survey for a response rate of 23.3% (AYA) and 19.0% (parents/caregivers). Twelve (44.4%) AYA were under age 18. Twenty-one (77.8%) AYA identified as female, 6 (22.2%) as male, and 0 (0%) as “other.” Self-reported diagnoses, confirmed by medical chart review, were categorized as sex chromosome DSD (11.1%), 46,XX DSD (29.6%), and 46,XY DSD (59.3%). Of parents/caregivers, 19 (86.4%) identified as female, 3 (13.6%) as male, and 0 (0%) as “other.” Additional demographic information is summarized Table 1. The study protocol was approved by the respective medical centers’ institutional review boards.

**Table 1:** Demographics of the adolescents/young adults and parent participants

Characteristics of the AYA	Self-reported by AYA (n = 27)	Parent-reported (n = 22)	Diagnosis excerpted from medical record
*Age of AYA*			
17 years or under	12 (44.4%)	11 (50%)	
18 years or older	15 (55.6%)	10 (45.5%)	
Unknown	0	1 (4.5%)	
*Diagnosis of AYA*			
Sex chromosome DSD	3 (11.1%)	3 (13.6%)	• 45,X/46,XY
			• Ovotesticular DSD
46,XX DSD	8 (29.6%)	8 (36.4%)	• 21-OH CAH
			• Ovotesticular DSD
			• Mayer-Rokitansky-Küster-Hauser syndrome
46,XY DSD	16 (59.3%)	11 (50.0%)	• 17 beta hydroxysteroid dehydrogenase 3 deficiency
			• Bilateral UDT, proximal hypospadias with chordee of unknown genetic etiology
			• Cloacal exstrophy
			• Complete androgen insensitivity syndrome
			• Complete gonadal dysgenesis
			• Epispadias
			• Frasier syndrome with gonadal dysgenesis
			• Partial androgen insensitivity syndrome
			• SF1 variant – partial gonadal dysgenesis
*Gender of AYA/parent*			
Female	21 (77.8%)	19 (86.4%)	
Male	6 (22.2%)	3 (13.6%)	
Other	0 (0%)	0 (0%)	
*Race of AYA/parent*			
White	25 (92.6%)	22 (100%)	
Non-white	2 (7.4%)	0 (0%)	
*Education level of parent*			
Less than high school		1 (4.5%)	
Some college or 2-year college degree		8 (36.4%)	
4-Year college degree or higher (Master’s Degree, PhD, etc.)		13 (59.1%)	

### Measures

The semantic differential tool measures the connotative meaning of concepts, terms, objects, events, etc. (Osgood, 1952; Osgood & Suci, 1955; Osgood et al., 1957; Ploder & Eder, 2015). Ratings of the topic of interest across sets of bipolar adjective scales show trends in a population’s perception of the topic (Osgood et al., 1957; Ploder & Eder, 2015). This tool has been used to measure perceptions of illnesses, including self-perceptions of individuals with these illnesses (Chotai & Eisemann, 1994; Devins et al., 2015; Fitzgerald et al., 2008; Molinari & Riva, 1995). It has also been used to measure perceptions from the perspective of caregivers (Chotai & Eisemann, 1994). Conditions that have been assessed using the semantic differential are often impacted by perceived social stigma (Chotai & Eisemann, 1994; Devins et al., 2015; Fitzgerald et al., 2008; Molinari & Riva, 1995). Because much has been written about the personal meaning and relationship to self-concept of terminology used by the medical community, we selected a semantic scaling method shown to tap self-concept. Internal self-esteem, as described in the semantic differential method used in the current study (Prescott, 1978), relates to an individual's feelings of efficacy and competence, which are derived from their perceptions of the influence they exert on their environment. Conversely, external self-esteem pertains to the evaluations and judgments of significant individuals within a person’s social circle. This method, which employs bipolar adjectives identified through factor analysis to distinguish between these self-esteem domains, is rooted in the original measure (Franks & Marolla, 1976). The 7 adjective pairs reflecting external self-esteem include: good-bad, nice-awful, generous-greedy, pleasant-unpleasant, dependable-undependable, honest-dishonest, and active–passive. The 8 adjective pairs comprising the internal self-esteem scale include: strong–weak, leader–follower, powerful-powerless, confident-unsure, curious-not curious, inventive-not inventive, sharp-dull, and active–passive. The “active–passive” scale is grouped with both dimensions because foundational work on the semantic differential demonstrated its universality irrespective of the particular concept being measured (Osgood, 1952; Osgood & Suci, 1955).

### Procedure

AYA (and a parent/caregiver) were mailed a cover letter from their primary subspecialty physician, accompanied by an invitation letter from the investigators. If interested in participating, participants were asked to email the investigators (SS or LW). The research team attempted to contact those who did not respond to the invitation after 10 days and up to three times by telephone. Participating families received links to two online surveys utilizing a Qualtrics® platform: one survey to be completed by the AYA and one by the parent/caregiver. Responses were linked for analysis by an assigned five-digit code. Both surveys contained the semantic rating scale, a measure of AYA global self-worth, and a health-related quality of life measure (not further discussed in this report). Participants were offered $20 as compensation for their effort.

**Fig. 2:**
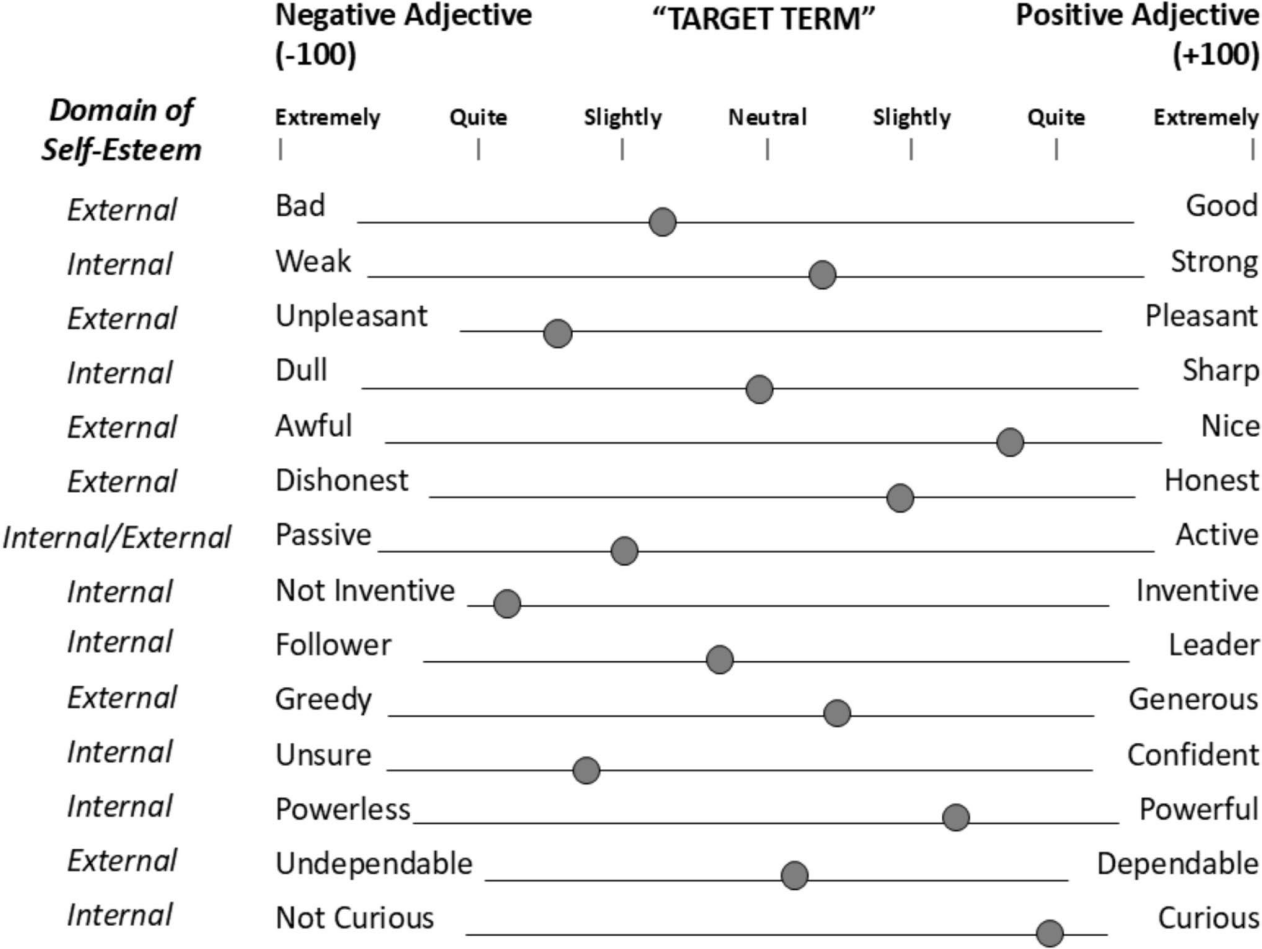
Depiction of the Franks-Marolla semantic differential of self-esteem

Participants are instructed to rate each specified term on the 14 bipolar adjectives using a visual analogue scale (− 100 for the negative pole to + 100 for the positive pole), with verbal anchors provided (Funke & Reips, 2012) (Fig. 2). The semantic differential overall has also been shown to have high item and factor-score reliability, as well as high face validity (Osgood, 1964). To validate the semantic differential measure of self-esteem in the current study—“Yourself As You Are Now”—the 5-item measure Global Self-Worth (GSW) scale adapted from the Self-Perception Profile (Harter, 2012, 2016), was also administered. Higher GSW scores indicate increasing agreement with more positive (than negative) self-statements. The GSW demonstrates high internal consistency based on 4 samples, with an average Cronbach’s α of 0.81, and has been shown to have strong validity. A Pearson correlation coefficient calculated between the GSW and “Yourself as You Are Now” scale scores was moderately correlated (*r* = 0.52), providing evidence of concurrent validity of the semantic differential measure.

Terms assessed using the semantic differential were presented to participants in a predetermined order. Following assent (or consent) to participate and demographic questions, “Asthma,” was presented as a sample semantic differential term to orient the participant to the methodology. The rest of the terms were then presented as follows: “Yourself as You Are Now” (“Your Child as They Are Now”), “Your Medical Condition” (“Your Child’s Medical Condition”), the term “Disorder of Sex Development,” and the term “Intersex.” Participants were asked to name or describe their/their child’s medical condition prior to rating “Your”/“Your Child’s Medical Condition.” The AYA reported the age at which they first learned about their medical condition, and the parent similarly reported the age at which their child had been informed. Definitions for “Disorder of Sex Development” and “Intersex” from the Consensus Statement and Human Rights Watch Report were also provided at the start of the semantic differential for each term, respectively (Human Rights Watch, 2017; Lee et al., 2006).

### Data Analysis

An arithmetic mean was calculated for the 7 bipolar adjective pairs representing external self-esteem and the 8 pairs reflecting internal self-esteem. Accordingly, each of the 4 target terms (“Yourself”/“Your Child as You/They are Now,” “Your/Your Child’s Medical Condition,” “Disorder of Sex Development,” and “Intersex”) yielded separate external and internal self-esteem scores for the AYA and participating parent. AYA and parent responses were analyzed independently. Comparisons among terms scores were made using Cohen’s *d* for effect size and paired *t*-tests (within AYA and parent groups).

## Results

### Term Comparisons

**Fig. 3:**
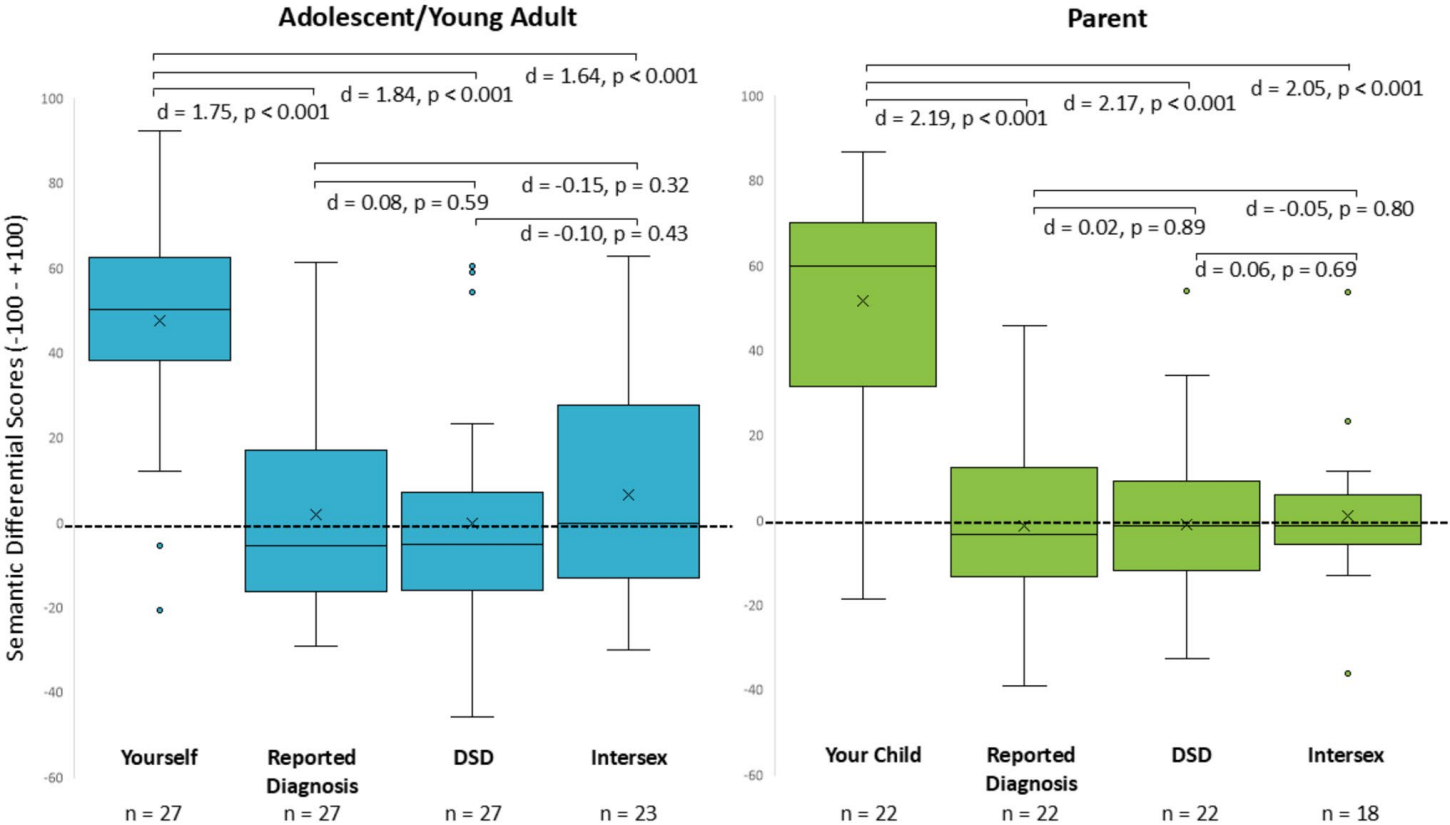
Box plots for the aggregated AYA and parent semantic differential scores for each of the 4 terms. Lower and upper box boundaries 25th and 75th percentiles, respectively; line inside box median; X inside box mean; lower and upper error lines 10th and 90th percentiles, respectively; filled circles data falling outside 10th and 90th percentiles. Abbreviation: AYA = adolescent or young adult

Box plots of AYA and parent semantic differential scores (external and internal scales, combined) for “Yourself”/“Your Child,” the reported diagnosis/phenotype, “Disorder of Sex Development (DSD),” and “Intersex” are illustrated in Fig. 3. Both AYA and Parents rated “Yourself”/“Your Child” most positively (*M* = 47.63 for “Yourself” and *M* = 51.71 for “Your Child”). The reported diagnosis/phenotype, “DSD,” and “Intersex” were all rated as relatively neutral (AYA: *M* = 1.89, − 0.19, 6.83, respectively; Parents: *M* = − 1.34, − 0.93, 1.12, respectively). Both AYA and Parents scored “Yourself”/“Your Child” significantly more positive than the reported diagnosis (AYA and Parents: *p* < 0.001), “DSD” (AYA and Parents: *p* ≤ 0.001), and “Intersex” (AYA and Parents: *p* < 0.001). There were large effect size differences between these means (respectively, AYA: *d* = 1.75, 1.84, 1.64; Parents: *d* = 2.19, 2.17, 2.05). Differences between the reported diagnosis, “DSD” and “Intersex” were not statistically significant for AYA or Parents (*p* > 0.05 for all comparisons) and effect size differences were small (*d* < 0.2 for all comparisons).

### External vs. Internal Self-Esteem

**Fig. 4:**
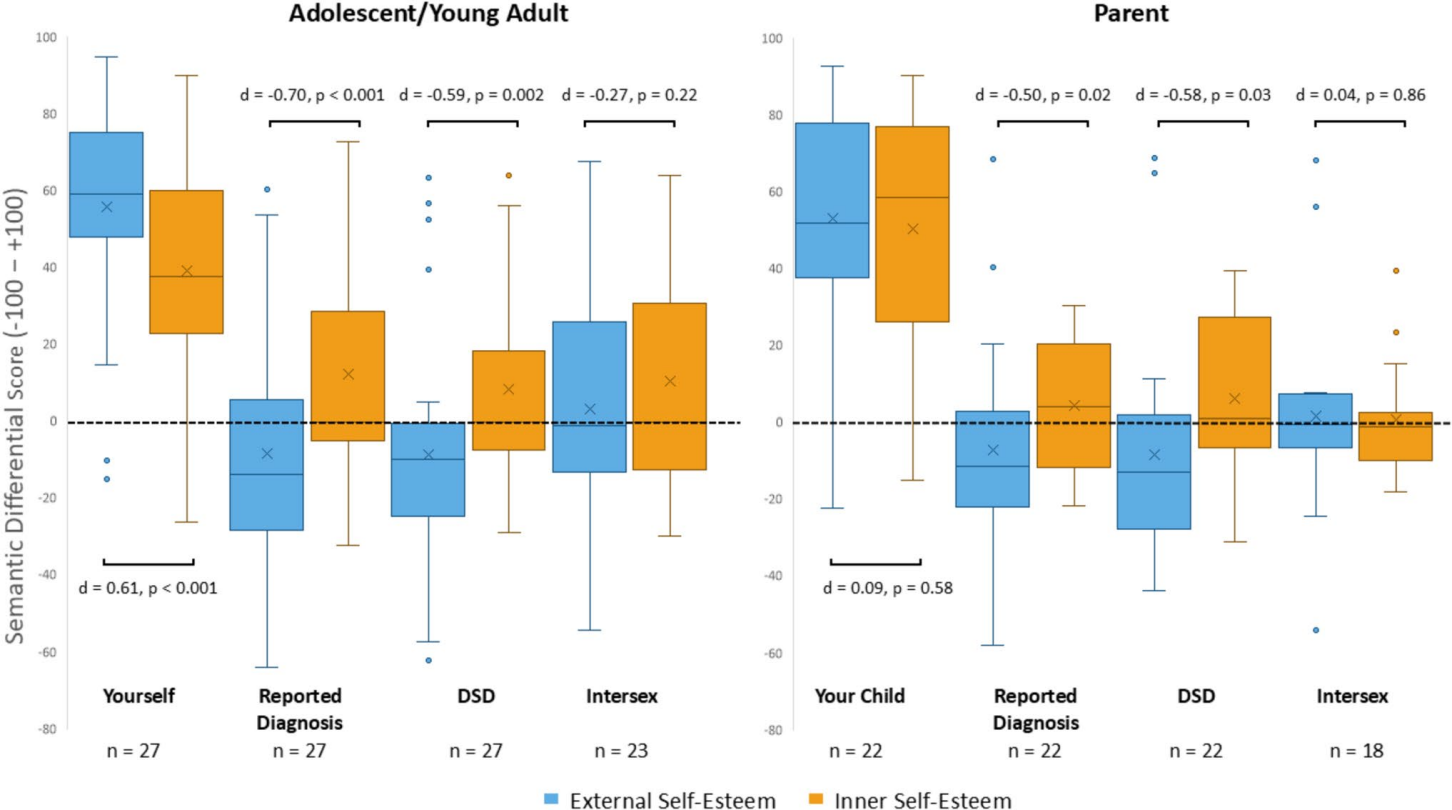
Box plots of AYA and parent semantic differential external and internal self-esteem scores for each of the 4 terms. Lower and upper box boundaries 25th and 75th percentiles, respectively, line inside box median, X inside box mean, lower and upper error lines 10th and 90th percentiles, respectively, filled circles data falling outside 10th and 90th percentiles. Abbreviation: AYA, adolescent or young adult

Figure 4 illustrates AYA and Parent semantic differential scores stratified by self-esteem domain (external and internal). The ordering of means revealed that AYA rated the reported diagnosis, “DSD,” and “Intersex” more negatively on the domain of external self-esteem (*M* = − 8.35, − 8.65, 3.07, respectively) compared to internal self-esteem (*M* = 12.14, 8.28, 10.58, respectively). The difference between domains was statistically significant only for the reported diagnosis and “DSD” (reported diagnosis: *p* < 0.001,* d* = − 0.70; “DSD”: *p* = 0.002, *d* = − 0.59). AYA rated “Yourself” significantly more positively on external self-esteem (*M* = 55.98) compared to internal self-esteem (*M* = 39.28) (*p* < 0.001,* d* = 0.61).

Parents rated reported diagnosis on the external self-esteem scale significantly more negative (*M* = − 7.06) than on the internal self-esteem scale (*M* = 4.83) (*p* = 0.02, *d* = − 0.50). The same was true for the ratings of “DSD” (− 8.21 vs 6.35 for external and internal self-esteem, respectively) (*p* = 0.03, *d* = − 0.58). The difference between parent ratings by domain of self-esteem for “Your Child” (External: *M* = 53.09; Internal: *M* = 50.32) and “Intersex” (External: *M* = 1.59; Internal: *M* = 0.65) were not statistically significant.

### Comparisons Between Medical Centers

To determine if responses were peculiar to those participating at a particular recruitment site, semantic differential scores were compared between the primary medical center (Site A) and collaborating medical centers (Sites B & C). Sites B and C were combined because of the small numbers recruited at the latter site. Across all 4 terms (“Yourself”/“Your Child,” the reported diagnosis, “DSD,” and “Intersex”) the order of mean ratings of both AYA and Parents from Site A were more negative than ratings of AYA and Parents from Sites B & C (respectively, AYA Site A: *M* = 44.66, − 5.15, − 9.69, − 2.5; AYA Sites B & C: *M* = 50.82, 9.48, 10.05, 14; Parents Site A: *M* = 50.88, − 8.77, − 6.10, − 1.81; Parents Sites B & C: *M* = 53.47, 14.59, 10.13, 5.72) (data not shown). This trend notwithstanding, the more negative ratings by AYA and parents at Site A than Sites B & C were not statistically significant, with the exceptions of AYA scores for “DSD” and Parent scores for the reported diagnosis (*p* < 0.05 for both).

### Age at Diagnosis and Time Since Diagnosis

AYA age at diagnosis (0–9 years vs. 10 + years) or time since diagnosis (0–12 years vs. 13–22 years) did not significantly differentiate semantic differential scores.

## Discussion

Prior to the International Consensus Conference on Intersex (Lee et al., 2006), terminology used by the medical community to categorize conditions affecting somatic sex determination and differentiation was based on gonadal history (i.e., “true hermaphroditism” and the sex-qualified subcategories of male/female pseudohermaphroditism). In interactions with patients, “intersexuality,” a term first introduced in the early 1900’s (Goldschmidt, 1917), became the preferred umbrella for all conditions that, post-consensus conference, were referred to as “disorders of sex development.” Although the new term was initially embraced by members of the intersex advocacy movement (Dreger et al., 2005), it was quickly rejected post Consensus Statement.

The current pilot study is the first to apply an established methodology—first introduced in the mid-1950’s and with extensive validation—to differentiate the meanings attributed to DSD-related terminology. In doing so, it suggests an alternative to understanding terminology beyond simple tests of preference for one term over another, which appears related to the particular stakeholder group targeted (D'Oro et al., 2020; Lin-Su et al., 2015; Tiryaki et al., 2018). Semantic scaling addresses several limitations of the existing literature by providing quantitative, implicit measurements of objects, events and concepts, in this case the person themself, their specific diagnosis (or phenotype), and the umbrella terms DSD and intersex.

Overall, study participants (AYA and parents) rated self/their child in a positive direction. The moderately correlated semantic differential scores for self with AYA-reported GSW scores reinforces interpretation of the rating scale as an index of self-concept. In contrast to ratings of yourself/your child, participants rated the specific diagnosis/phenotype, “Disorder of Sex Development,” and “Intersex” very similarly and far more negatively. There were no significant differences between the perceptions of these three terms and all were easily differentiated from the ratings for “You”/“Your Child.” This observation, at least for this limited group of participants, challenges the suggestion that individuals with a DSD personally "identify" with the various terms used to describe their condition (Davis, 2013; Feder & Karkazis, 2008). If this finding can be generalized to a larger and more diverse sample, it would support one of the benefits hoped for by intersex advocates around the time of the Consensus Statement: that the new nomenclature "DSD" would "label the condition rather than the person" (Dreger et al., 2005; Intersex Society of North America, n.d.-a; n.d.-b).

The use of the modified semantic differential tool adopted for this study (Franks & Marolla, 1976; Prescott, 1978) facilitated a deeper dive into the meaning associated with each term using bipolar adjectives that have been used to characterize the constructs of external and internal self-esteem. AYA rated their external self-esteem significantly more positively than internal self-esteem. In contrast, ratings by both AYA and parents showed a reverse pattern when the term to be rated was the reported diagnosis and DSD, i.e., both terms were associated with significantly more positive internal than external scores. The importance of these differences remains to be evaluated in future studies. A qualitative methods study by Lundberg et al. (2021) illustrated that participants have diverse preferences for terminology based on their personal experience and the social context; many have developed an understanding of which terms are alienating and which terms elicit support from others in particular contexts.

Notwithstanding the novel methodological approach and findings of this study, which challenge the way we think about DSD terminology, several limitations with implications for the generalizability of the findings must be taken into account. First, the sample sizes for both the AYA and parent cohorts were small and the participation rate low. Further, the educational attainment of participating parents (59% having received a bachelor’s degree) is substantially higher than that in the general population [36% as reported by the US Census for 2019 (United States Census Bureau, 2020)]. This pilot study was conducted to learn if semantic differential methodology could be used to reframe the question about preference over various terms to an investigation of the differential meanings ascribed to each. We have no expectation that the semantic differential scores will necessarily be replicated in a study employing a larger and more demographically diverse sample. In point of fact, when participant scores across sites were compared, there were differences in absolute scores for each term; however, the overall pattern of results remained unchanged. Specifically, the term you/your child was rated more positively than all condition-related terms, which were rated very similarly.

Further explorations into the implicit perceptions and contextual preferences of terminology will provide new insights to guide providers and researchers working to enhance communication among clinicians, researchers, patients and their caregivers. Language, as a reflection of societal attitudes, has the power to stigmatize and disempower, to minimize autonomy where it should be maximized, and to reduce a person to a singular category when other facets of their identity should not be overlooked (Coleman Brown, 2013; Dunn & Andrews, 2015). At the same time, language can also serve as a powerful tool to empower individuals to counteract oppression and marginalization and promote a sense of community and identity (Dunn & Andrews, 2015). Ultimately, developing sensitivity for the connotative meanings individuals place on terminology used to describe medical conditions or differences, more generally, will enable the medical and research communities to better address this key element of patient-centered care.

## Data Availability

All data generated or analyzed during this study are included in this article. Further inquiries can be directed to the corresponding author.

## References

[CR1] Ahmed, S. F., Achermann, J. C., Arlt, W., Balen, A. H., Conway, G., Edwards, Z. L., Elford, S., Hughes, I. A., Izatt, L., Krone, N., Miles, H. L., O’Toole, S., Perry, L., Sanders, C., Simmonds, M., Wallace, A. M., Watt, A., & Willis, D. (2011). UK guidance on the initial evaluation of an infant or an adolescent with a suspected disorder of sex development. *Clinical Endocrinology,**75*(1), 12–26. 10.1111/j.1365-2265.2011.04076.x 21521344 10.1111/j.1365-2265.2011.04076.x PMC3132446

[CR2] Bennecke, E., Köhler, B., Rohle, R., Thyen, U., Gehrmann, K., Lee, P., Nordenström, A., Cohen-Kettenis, P., Bouvattier, C., Wiesemann, C., & on behalf of dsd-LIFE group. (2021). Disorders or differences of sex development? Views of affected individuals on DSD terminology. *Journal of Sex Research,**58*(4), 522–531. 10.1080/00224499.2019.1703130 31985272 10.1080/00224499.2019.1703130

[CR3] Chotai, J., & Eisemann, M. (1994). Perception of spouse in relation to perception of self by semantic differentials in depressed patients and their spouses. *Acta Psychiatrica Scandinavica,**90*(2), 114–119. 10.1111/j.1600-0447.1994.tb01565.x 7976456 10.1111/j.1600-0447.1994.tb01565.x

[CR4] Coleman Brown, L. (2013). Stigma: An enigma demystified. In L. J. Davis (Ed.), *The disability studies reader* (pp. 147–160). Taylor & Francis Group. https://lccn.loc.gov/2016013306

[CR5] D’Oro, A., Rosoklija, I., Jacobson, D. L., Finlayson, C., Chen, D., Tu, D. D., Austin, P. F., Karaviti, L. P., Gunn, S. K., Nahata, L., Kapa, H. M., Kokorowski, P. J., Kim, M. S., Messina, A. C., Kolon, T. F., Yerkes, E. B., Cheng, E. Y., & Johnson, E. K. (2020). Patient and caregiver attitudes toward disorders of sex development nomenclature. *Journal of Urology,**204*(4), 835–842. 10.1097/JU.0000000000001076 32302259 10.1097/JU.0000000000001076

[CR6] Davies, J. H., Knight, E. J., Savage, A., Brown, J., & Malone, P. S. (2011). Evaluation of terminology used to describe disorders of sex development. *Journal of Pediatric Urology,**7*(4), 412–415. 10.1016/j.jpurol.2010.07.004 20708971 10.1016/j.jpurol.2010.07.004

[CR7] Davis, G. (2013). The power in a name: Diagnostic terminology and diverse experiences. *Psychology & Sexuality,**5*(1), 15–27. 10.1080/19419899.2013.831212

[CR8] Devins, G. M., Wong, J. C., Payne, A. Y., Lebel, S., Lee, R. N., Mah, K., Irish, J., & Rodin, G. (2015). Distancing, self-esteem, and subjective well-being in head and neck cancer. *Psycho-Oncology,**24*(11), 1506–1513. 10.1002/pon.3760 25631628 10.1002/pon.3760

[CR9] Dreger, A. D., Chase, C., Sousa, A., Gruppuso, P. A., & Frader, J. (2005). Changing the nomenclature/taxonomy for intersex: A scientific and clinical rationale. *Journal of Pediatric Endocrinology and Metabolism,**18*(8), 729–733. 10.1515/jpem.2005.18.8.729 16200837 10.1515/jpem.2005.18.8.729

[CR10] Dunn, D. S., & Andrews, E. E. (2015). Person-first and identity-first language: Developing psychologists’ cultural competence using disability language. *American Psychologist,**70*(3), 255–264. 10.1037/a0038636 25642702 10.1037/a0038636

[CR11] Feder, E. K., & Karkazis, K. (2008). What’s in a name? The controversy over “disorders of sex development.” *Hastings Center Report,**38*(5), 33–36. 10.1353/hcr.0.0062 18947138 10.1353/hcr.0.0062

[CR12] Fitzgerald, J. T., Stansfield, R. B., Tang, T., Oh, M., Frohna, A., Armbruster, B., Gruppen, L., & Anderson, R. (2008). Patient and provider perceptions of diabetes: Measuring and evaluating differences. *Patient Education and Counseling,**70*(1), 118–125. 10.1016/j.pec.2007.09.005 17997265 10.1016/j.pec.2007.09.005 PMC2223066

[CR13] Franks, D. D., & Marolla, J. (1976). Efficacious action and social approval as interacting dimensions of self-esteem: A tentative formulation through construct validation. *Sociometry,**39*(4), 324–341. 10.2307/3033498

[CR14] Funke, F., & Reips, U.-D. (2012). Why semantic differentials in web-based research should be made from visual analogue scales and not from 5-point scales. *Field Methods,**24*(3), 310–327. 10.1177/1525822x12444061

[CR15] Gluyas, H. (2015). Patient-centred care: Improving healthcare outcomes. *Nursing Standard,**30*(4), 50–57. 10.7748/ns.30.4.50.e10186 26394978 10.7748/ns.30.4.50.e10186

[CR16] Goldschmidt, R. (1917). Intersexuality and the endocrine aspect of sex. *Endocrinology,**1*(4), 433–456. 10.1210/endo-1-4-433

[CR17] Harter, S. (2012). *Manual for the self-perception profile for adolescents: Manual and questionnaires*. University of Denver.

[CR18] Harter, S. (2016). *The self-perception profile for emerging adults: Manual and questionnaire*. University of Denver.

[CR19] Human Rights Watch. (2017). *“I want to be like nature made me.” Medically unnecessary surgeries on intersex children in the US*. https://www.hrw.org/sites/default/files/report_pdf/lgbtintersex0717_web_0.pdf

[CR20] Institute of Medicine Committee on Quality of Health Care in America. (2001). *Crossing the quality chasm: A new health system for the 21st century*. National Academies Press. 10.17226/10027 25057539

[CR21] Intersex Society of North America. (n.d.-a). *What are disorders of sex development?* Retrieved December 7, 2024 from https://isna.org/node/1028/#what-are-disorders-of-sex-development

[CR22] Intersex Society of North America. (n.d.-b). *Why is ISNA using DSD?* Retrieved December 7, 2024 from https://isna.org/node/1066/

[CR23] Johnson, E. K., Rosoklija, I., Finlayson, C., Chen, D., Yerkes, E. B., Madonna, M. B., Holl, J. L., Baratz, A. B., Davis, G., & Cheng, E. Y. (2017). Attitudes towards “disorders of sex development” nomenclature among affected individuals. *Journal of Pediatric Urology,**13*(6), 601–608. 10.1016/j.jpurol.2017.03.035 10.1016/j.jpurol.2017.03.035 28545802

[CR24] Kreukels, B. P. C., Cohen-Kettenis, P. T., Roehle, R., van de Grift, T. C., Slowikowska-Hilczer, J., Claahsen-van der Grinten, H., Linden Hirschberg, A., de Vries, A. L. C., Reisch, N., Bouvattier, C., Nordenstrom, A., Thyen, U., Kohler, B., & On behalf of the dsd-LIFE group. (2019). Sexuality in adults with differences/disorders of sex development (DSD): Findings from the dsd-LIFE study. *Journal of Sex & Marital Therapy,**45*(8), 688–705. 10.1080/0092623X.2019.1610123 10.1080/0092623X.2019.1610123 31034334

[CR25] Lee, P. A., Houk, C. P., Ahmed, S. F., Hughes, I. A., International Consensus Conference on Intersex organized by the Lawson Wilkins Pediatric Endocrine Society, & the European Society for Paediatric Endocrinology. (2006). Consensus statement on management of intersex disorders. *Pediatrics,**118*(2), 488–500. 10.1542/peds.2006-0738 10.1542/peds.2006-0738 16882788

[CR26] Lin-Su, K., Lekarev, O., Poppas, D. P., & Vogiatzi, M. G. (2015). Congenital adrenal hyperplasia patient perception of “disorders of sex development” nomenclature. *International Journal of Pediatric Endocrinology,**2015*(1). 10.1186/s13633-015-0004-4 10.1186/s13633-015-0004-4 PMC4360949 25780368

[CR27] Lines, L. M., Lepore, M., & Wiener, J. M. (2015). Patient-centered, person-centered, and person-directed care: They are not the same. *Medical Care,**53*(7), 561–563. 10.1097/MLR.0000000000000387 26067878 10.1097/MLR.0000000000000387

[CR28] Lundberg, T., Hegarty, P., & Roen, K. (2018). Making sense of ‘intersex’ and ‘DSD’: How laypeople understand and use terminology. *Psychology & Sexuality,**9*(2), 161–173. 10.1080/19419899.2018.1453862

[CR29] Lundberg, T., Roen, K., Kraft, C., & Hegarty, P. (2021). How young people talk about their variations in sex characteristics: Making the topic of intersex talkable via sex education. *Sex Education-Sexuality Society and Learning*. 10.1080/14681811.2021.1911796

[CR30] Miller, L., Leeth, E. A., Johnson, E. K., Rosoklija, I., Chen, D., Aufox, S. A., & Finlayson, C. (2018). Attitudes toward “disorders of sex development” nomenclature among physicians, genetic counselors, and mental health clinicians. *Journal of Pediatric Urology,**14*(5), 411–418. 10.1016/j.jpurol.2018.08.009 10.1016/j.jpurol.2018.08.009 30224300

[CR31] Molinari, E., & Riva, G. (1995). Self-others perception in a clinical sample of obese women. *Perceptual and Motor Skills,**80*(3 Pt 2), 1283–1289. 10.2466/pms.1995.80.3c.1283 7478889 10.2466/pms.1995.80.3c.1283

[CR32] Osgood, C. E. (1952). The nature and measurement of meaning. *Psychological Bulletin,**49*(3), 197–237. 10.1037/h0055737 14930159 10.1037/h0055737

[CR33] Osgood, C. E. (1964). Semantic differential technique in the comparative study of cultures. *American Anthropologist,**66*(3), 171–200. 10.1525/aa.1964.66.3.02a00880

[CR35] Osgood, C. E., & Suci, G. J. (1955). Factor analysis of meaning. *Journal of Experimental Psychology,**50*(5), 325–338. 10.1037/h0043965 13271697 10.1037/h0043965

[CR34] Osgood, C. E., Suci, G., & Tannenbaum, P. (1957). *The measurement of meaning*. University of Illinois Press.

[CR36] Pasterski, V., Prentice, P., & Hughes, I. A. (2010). Consequences of the Chicago consensus on disorders of sex development (DSD): Current practices in Europe. *Archives of Disease in Childhood,**95*(8), 618–623. 10.1136/adc.2009.163840 19773218 10.1136/adc.2009.163840

[CR37] Ploder, A., & Eder, A. (2015). Semantic differential. In J. D. Wright (Ed.), *International encyclopedia of the social & behavioral sciences* (pp. 563–571). Elsevier. 10.1016/b978-0-08-097086-8.03231-1

[CR38] Prescott, P. A. (1978). Sex differences on a measure of self-esteem: Theoretical implications. *Journal of Genetic Psychology,**132*, 67–85. 10.1080/00221325.1978.10533316 650183 10.1080/00221325.1978.10533316

[CR39] Reis, E. (2007). Divergence or disorder?: The politics of naming intersex. *Perspectives in Biology and Medicine,**50*(4), 535–543. 10.1353/pbm.2007.0054 17951887 10.1353/pbm.2007.0054

[CR40] Tiryaki, S., Tekin, A., Yagmur, I., Ozen, S., Ozbaran, B., Goksen, D., Darcan, S., Ulman, I., & Avanoglu, A. (2018). Parental perception of terminology of disorders of sex development in western Turkey. *Journal of Clinical Research in Pediatric Endocrinology,**10*(3), 216–222. 10.4274/jcrpe.0007 29595517 10.4274/jcrpe.0007 PMC6083470

[CR41] United States Census Bureau. (2020). *U.S. Census Bureau releases new educational attainment data*. Retrieved June 18, 2021 from https://www.census.gov/newsroom/press-releases/2020/educational-attainment.html

[CR42] Whitehead, J., Adams, M., Davies, A., Johnson, E. K., & Pyle, L. T. C. (2025). Evolving language reflects evolving understanding: Updated terms for anatomical description in i/VSC/DSD. *Nature Reviews Urology*. 10.1038/s41585-025-01035-5 40229428 10.1038/s41585-025-01035-5

[CR43] Wiesemann, C., Ude-Koeller, S., Sinnecker, G. H., & Thyen, U. (2010). Ethical principles and recommendations for the medical management of differences of sex development (DSD)/intersex in children and adolescents. *European Journal of Pediatrics,**169*(6), 671–679. 10.1007/s00431-009-1086-x 19841941 10.1007/s00431-009-1086-x PMC2859219

[CR44] Wisniewski, A. B., Batista, R. L., Costa, E. M. F., Finlayson, C., Sircili, M. H. P., Denes, F. T., Domenice, S., & Mendonca, B. B. (2019). Management of 46,XY differences/disorders of sex development (DSD) throughout life. *Endocrine Reviews,**40*(6), 1547–1572. 10.1210/er.2019-00049 31365064 10.1210/er.2019-00049

[CR45] Wit, J. M., Ranke, M. B., Kelnar, C. J. H. (eds.). (2007). ESPE classification of paediatric endocrine diagnoses. *Hormone Research*, *68*(Supp. l2), 1–119.

